# Self-Supervised Action Representation Learning Based on Asymmetric Skeleton Data Augmentation

**DOI:** 10.3390/s22228989

**Published:** 2022-11-20

**Authors:** Hualing Zhou, Xi Li, Dahong Xu, Hong Liu, Jianping Guo, Yihan Zhang

**Affiliations:** 1 College of Information Science and Engineering, Hunan Normal University, Changsha 410081, China; 2 Key Laboratory of Sports Intelligence Reasearch, Hunan Normal University, Changsha 410081, China; 3 College of Physical Culture, Hunan Normal University, Changsha 410081, China

**Keywords:** action representation, contrastive learning, data augmentation, self-supervised

## Abstract

Contrastive learning has received increasing attention in the field of skeleton-based action representations in recent years. Most contrastive learning methods use simple augmentation strategies to construct pairs of positive samples. When using such pairs of positive samples to learn action representations, deeper feature information cannot be learned, thus affecting the performance of downstream tasks. To solve the problem of insufficient learning ability, we propose an asymmetric data augmentation strategy and attempt to apply it to the training of 3D skeleton-based action representations. First, we carefully study the different characteristics presented by different skeleton views and choose a specific augmentation method for a certain view. Second, specific augmentation methods are incorporated into the left and right branches of the asymmetric data augmentation pipeline to increase the convergence difficulty of the contrastive learning task, thereby significantly improving the quality of the learned action representations. Finally, since many methods directly act on the joint view, the augmented samples are quite different from the original samples. We use random probability activation to transform the joint view to avoid extreme augmentation of the joint view. Extensive experiments on NTU RGB + D datasets show that our method is effective.

## 1. Introduction

Due to the complexity of human actions, video-based representations of human actions have received increasing attention in the field of computer vision. With the popularity of depth sensors [1] and the development of pose estimation algorithms [2,3,4], it is possible to extract skeleton data with robustness in complex environments. Therefore, action representation algorithms based on skeleton data have received significant attention. However, most of the existing skeleton-based action representation algorithms [5,6,7,8,9] adopt supervised training methods, which require precise annotation of training samples, but this process is expensive and time-consuming. Self-supervised methods are increasingly showing their advantages, in which the information of unlabeled training samples themselves is used to learn action representations. Earlier methods have focused on exploiting the sample structural integrity of pretext tasks to learn action representations [10,11,12,13]. Unlike before, most recent methods are implemented based on the contrastive learning framework [14,15,16,17,18]. In these methods, with strong generalization through simple pretext tasks, some feature representations can be learned and easily extended to downstream tasks such as classification and recognition.

In action representation algorithms based on contrastive learning, data augmentation is one of the important components, which plays a crucial role in the performance of contrastive learning. Several studies [19,20] have shown that excellent data augmentation can obtain abundant semantic information, which can significantly improve the generalization ability of learned representations. However, an unsuitable data augmentation strategy will lead to a large difference between the augmented samples and the original data, which will affect the performance of the training results. In addition, the edges in the skeleton graph are fixed, and the joints represented by the graph nodes contain less information, so it is difficult for general data augmentation strategies to generate better pairs of positive samples. Therefore, a data augmentation strategy that can effectively improve the performance of contrastive learning needs to be explored and designed.

**Figure 1:**
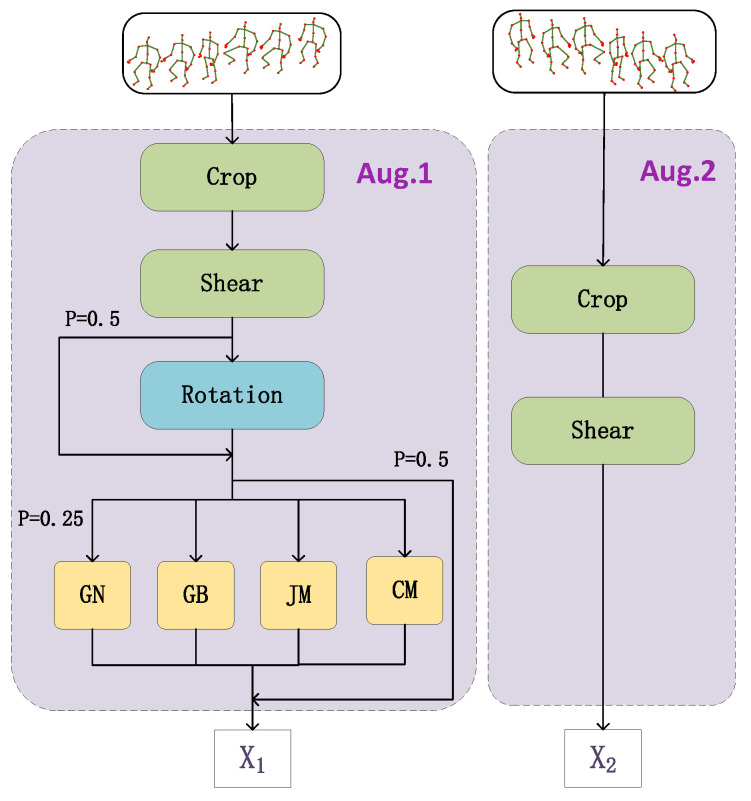
Asymmetric data augmentation pipeline.

Inspired by CrosSCLR [16], in this paper, a new data augmentation strategy is used to solve the above problems. An asymmetric data augmentation pipeline is designed, and the architecture is shown in Figure 1. The pipeline consists of two branches, left and right. In the left branch, seven basic data augmentation methods are integrated, and five methods, such as rotation and Gaussian blur, are randomly applied for probability activation. The two basic methods of crop and shear are integrated in the right branch. The advantage of this design is that it can not only ensure sufficient differences between the generated sample pairs but also avoid the generation of extreme samples. First, a skeleton sequence is input to the left and right branches of the asymmetric augmentation pipeline. After being processed by various augmentation methods in the branches, a pair of good sample pairs $x_{1}$ and $x_{2}\;$ is generated for subsequent learning tasks. Under the premise of avoiding excessively distorted samples, the data augmentation strategy designed in this paper is applied to the skeleton graph, which can incorporate as many data augmentation methods as possible. This strategy increases the difference between pairs of samples, thereby improving the convergence difficulty of the contrastive learning task and reducing the distribution shift between self-supervised pre-training and supervised fine-tuning caused by extreme augmentation. Representations learned through this strategy are highly robust to semantically irrelevant variations, further improving the performance of contrastive learning.

The main contributions of this paper are as follows:

1. Each view describes different types of skeleton data. We use specific data augmentation methods for each view according to the characteristics of different views and combine these methods.
2. We propose a new data augmentation strategy for skeleton sequences. An asymmetric augmentation pipeline with left and right branches is designed, where each branch is composed of different data augmentation methods.
3. We conduct extensive experiments on two large-scale 3D skeleton datasets (NTU RGB + D 60 and NTU RGB + D 120) to demonstrate the effectiveness of the proposed data augmentation strategy.

The overall structure of this article is summarized as follows. In Section 2, the mainstream methods of action representation in supervised learning are briefly introduced, and then the research progress of contrastive learning and the latest achievements of skeleton action representation based on contrastive learning are described. In Section 3, we focus on our asymmetric data augmentation strategy and apply it to the skeleton action representation framework of self-supervised single view and multiple views to improve the performance of the model. In order to evaluate the proposed method, we select a widely used evaluation protocol and present the results of the model on different datasets in Section 4. Finally, in Section 5, we present the conclusions of this research work and outline further development directions.

## 2. Related Work

In this section, we study the mainstream supervised action representation methods and learn about the shortcomings of supervised learning. At the same time, we study the contrastive learning method in self-supervised learning, and the skeleton action representation method based on contrastive learning. The research of these methods lays the foundation for the work in this paper.

### 2.1. Action Representation

Early skeleton-based action representation algorithms usually utilize handcrafted features [21,22,23] to model the geometric relationships between joints. Recent methods mainly focus on three aspects: (1) For the sequential structure of the skeleton, by using a recurrent neural network (RNN) [24,25,26], its temporal features can be more effectively utilized, but recurrent neural networks have the disadvantage of a vanishing gradient [27]. (2) Methods based on convolutional neural networks (CNNs) [5,28,29] first convert the skeleton sequence into a pseudo-image representation and use it as the input of the network, thereby transforming the action recognition into the image classification. (3) The method of graph convolutional networks (GCNs) constructs a spatio-temporal graph [6] to represent the 3D skeleton, and then uses graph convolution to simultaneously encode the temporal and spatial dimensions of the skeleton graph to better represent the temporal and spatial structures of action features. Some improved methods [7,30,31] incorporate an attention mechanism into the spatio-temporal graph to adaptively capture the associated features of the joints in the spatio-temporal space. Although these models achieve excellent performance in skeleton-based action recognition, they rely on expensive action sequence annotations.

### 2.2. Self-Supervised Action Representation

In self-supervised methods, unlabeled data are used to learn feature representations. Generally, pretext tasks are designed to generate supervision, and the quality of the pretext tasks affects the performance of the model. In the last few years, numerous self-supervised representation learning works based on contrastive learning have emerged, such as MoCo [17], MoCo v2 [18], SimCLR [32], BYOL [33], contrastive cluster [34], DINO [35], and SimSiam [36]. These methods show the same or even better performance than supervised methods in downstream tasks. For example, MoCo constructs a pair of positive samples and a dynamic queue of negative samples for contrastive learning. Inspired by SimCLR, MoCo v2 adds an MLP projection head and a more complex data augmentation method on the basis of MoCo to achieve better performance. In this paper, we follow the MoCo v2 framework to implement our method.

In action representation, the contrastive learning method has also been gradually introduced to improve the performance of the algorithms. A momentum encoder and a dynamic queue of negative samples are used for contrastive learning of skeleton sequences, while multiple data augmentation strategies are employed to learn skeleton features [15]. This method demonstrates the huge potential of self-supervised action representations. In MS2L [14], three tasks, namely, a motion prediction generation task, a jigsaw puzzle recognition task, and skeleton transformation-based contrastive learning, are integrated. An encoder-decoder structure with recurrent layers is designed to learn more general representations. This method solves the overfitting problem of learning skeleton representations in a single reconstruction task. AimCLR [37] proposes an extreme augmentation strategy for motion patterns that forces the model to learn more general representations by providing harder sample pairs. The method further explores data augmentation strategies. A new drop mechanism is used to solve the overfitting problem in self-supervised learning. ISC [38] uses both graph-based and sequence-based methods to describe skeleton data. The method learns skeleton features in a cross-contrastive manner and explores different skeleton-specific augmentation methods. CrosSCLR [16] proposes cross-view contrastive learning, which exploits the complementary information between views to mine positive sample pairs from similar negative samples to better extract skeleton features. This method solves the unreasonable problem of forcibly removing negative samples with a strong similarity to traditional contrastive learning.

## 3. Method

In this paper, the ST-GCN [6] block is used as the encoder, and MOCO v2 [18] is used as the basic framework for contrastive learning to optimize the encoder training. To improve the performance of contrastive learning, we designed an asymmetric data augmentation strategy for skeleton data. Our goal is to use the asymmetric augmentation strategy as a pretext task to make the results of contrastive learning more robust and achieve better performance in the downstream task of action recognition. In Section 3.1, we apply the asymmetric data augmentation strategy to a basic framework for action representation learning that uses single-view (joint) information for action representation learning. In Section 3.2, we apply the asymmetric data augmentation strategy to a composite framework for action representation, which uses multi-view (joint + motion) information and cross-view consistency knowledge mining to learn action representations. In Section 3.3, we focus on several typical basic augmentation methods used in the asymmetric augmentation strategy. In Section 3.4, we present a detailed description and theoretical analysis of the proposed asymmetric data augmentation strategy.

### 3.1. Basic Framework for Action Representation Based on Asymmetric Augmentation

**Figure 2:**
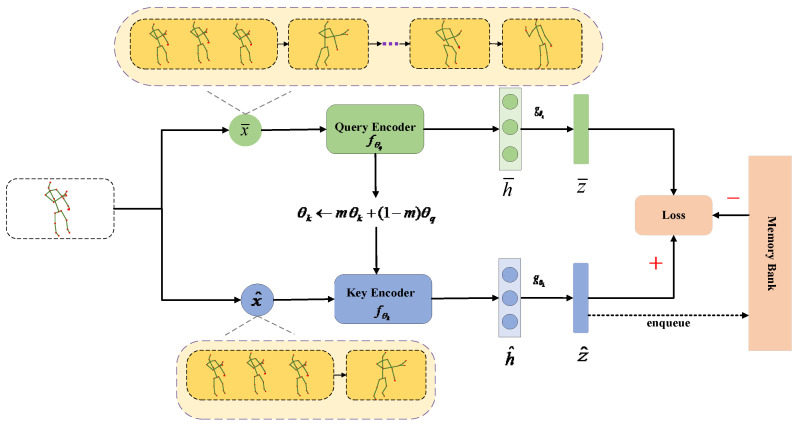
Basic framework based on asymmetric data augmentation strategy.

The basic framework based on the asymmetric augmentation strategy uses the information of a single-view (joint) to learn the feature representation. To aid in understanding, we describe the detailed training process of the proposed method in Algorithm 1. As shown in Figure 2, the basic framework is mainly composed of the following components:

1. Data augmentation: Skeleton sequences are randomly transformed into $\bar{x},\;\hat{x}$, as pairs of positive samples. Different data augmentation methods are combined in the left and right branches of the asymmetric augmentation pipeline, as shown in the yellow area in Figure 2.
2. Feature encoding: $\bar{x}$ and $\hat{x}$ are embedded into the hidden space by encoders $f_{\theta_{q}}$ and $f_{\theta_{k}}$: $\bar{h}=f_{\theta_{q}}\left(\bar{x}\right)$ and $\hat{h}=f_{\theta_{k}}\left(\hat{x}\right)$, where $\bar{h}$, $\hat{h}\in R^{c_{h}}.\;\;f_{\theta_{k}}$ is momentum updated by Equation (1),
  (1) $$\theta_{k}\leftarrow m\theta_{k}+\left(1-m\right)\theta_{q}$$
  where $\theta_{q}\;$ and $\theta_{k}\;$ are the parameters of encoders $f_{\theta_{q}}$ and $f_{\theta_{k}}$ respectively, and mϵ[0,1) is the momentum coefficient.
3. Nonlinear mapping: MLP projection heads $\;g_{\theta_{q}}$ and $g_{\theta_{k}}$ are used to map latent vectors $\bar{h}$ and $\hat{h}$ to the low-dimensional space: $\bar{z}=g_{\theta_{q}}\left(\bar{h}\right),\;\hat{z}=g_{\theta_{k}}\left(\hat{h}\right)$, $\bar{z}$,$\hat{z}\in R^{c_{z}}$.
4. Queue update: A queue $M={\left\{k_{\left(j\right)}\right\}}_{j=1}^{K}$ that stores a large number of negative samples is maintained to avoid redundant computation and iteratively updated by $\hat{z}$.
5. Contrast loss: InfoNCE [39] is used to train the network:

(2) $$L=-log\frac{\exp\left(z\cdot \hat{z}/\tau \right)}{\exp\left(z\cdot \hat{z}/\tau \right)+\sum_{i=1}^{M}\exp(z\cdot m_{i}/\tau )}$$

where τ is the temperature hyperparameter [40].
**Algorithm 1.** Main algorithm of Basic framework    **Input:** Temperature τ, momentum coefficient m, mini-batch size n, query encoder $f_{\theta_{q}}$, key encoder $f_{\theta_{k}}$, queue size K
    **Output:** The pre-trained encoder $f_{\theta_{q}}$.
    # Initialization
    Randomly initialize parameters $\theta_{q}$ of $f_{\theta_{q}}$, and copy to $f_{\theta_{k}}$ (parameters $\theta_{k}$)
    Randomly initialize negative keys ${\left\{k_{\left(j\right)}\right\}}_{j=1}^{K}$ in queue.
    **for** a sample mini-batch ${\left\{x_{\left(i\right)}\right\}}_{i=1}^{n}$ do 
        **for all**
iϵ{1,⋯,n}  do
              # Select asymmetric augmentation strategy to perform two random augments
              ${\bar{x}}_{\left(i\right)}=Aug1\left(x_{\left(i\right)}\right)$, ${\hat{x}}_{\left(i\right)}=Aug2\left(x_{\left(i\right)}\right)$
              # Feature encoding
              ${\bar{h}}_{\left(i\right)}=f_{\theta_{q}}\left({\bar{x}}_{\left(i\right)}\right)$, ${\hat{h}}_{\left(i\right)}=f_{\theta_{k}}\left({\hat{x}}_{\left(i\right)}\right)$
              # Nonlinear mapping
              ${\bar{z}}_{\left(i\right)}=g_{\theta_{q}}({\bar{h}}_{\left(i\right)}$), ${\hat{z}}_{\left(i\right)}=g_{\theta_{k}}\left({\hat{h}}_{\left(i\right)}\right)$
              detach ${\hat{z}}_{\left(i\right)}$
        **end for**
        # Calculate contrastive loss ℒ for mini-batch and update encoders                        $ℒ=-\frac{1}{n}\overset{n}{\underset{i=1}{\sum }}\log\frac{\exp\left({\bar{z}}_{\left(i\right)}\cdot {\hat{z}}_{\left(i\right)}/\tau \right)}{\exp\left({\bar{z}}_{\left(i\right)}\cdot {\hat{z}}_{\left(i\right)}/\tau \right)+\sum_{j=1}^{K}\exp\left({\bar{z}}_{\left(i\right)}\cdot k_{\left(j\right)}/\tau \right)}$
        Update $f_{\theta_{q}}\;$ to minimize ℒ
        Update $f_{\theta_{k}}$ with momentum: $\theta_{k}\leftarrow m\theta_{k}+\left(1-m\right)\theta_{q}$
        # Update queue
        Enqueue keys of current mini-batch ${\left\{{\hat{z}}_{\left(i\right)}\right\}}_{i=1}^{n}$
        Dequeue the oldest mini-batch of keys
    **end for**

### 3.2. Composite Framework for Action Representation Based on Asymmetric Augmentation

In the basic framework of contrastive learning, instance discrimination only uses a pair of positive samples, and the embeddings of other samples will be forcibly removed in the embedding space even if they have a high similarity with the embeddings of the original samples, which is unreasonable. To enable samples of the same class to be closely distributed in the embedding space, a multi-view optimized contrastive learning composite framework is proposed. The overall algorithm of the composite framework is shown in Algorithm 2. The views [30,41] of the skeleton can be easily obtained. Motion is represented as the temporal displacement between frames, and bone is the distance between two neighboring joints in the same frame. This paper uses three views: joint, motion, and bone.

**Figure 3:**
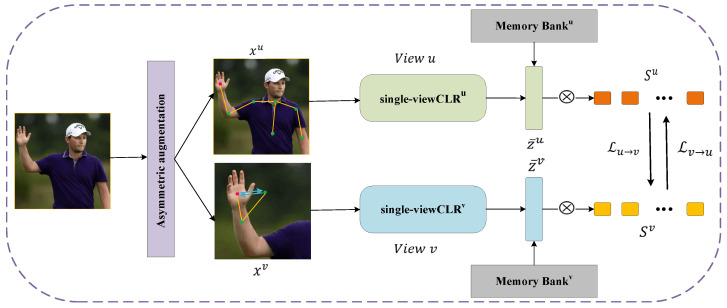
Composite framework based on asymmetric data augmentation strategy. ⊗ denotes the dot product.

Multi-view optimization utilizes the high similarity of samples in one view to guide the learning process in another view. Other positive samples are first mined using a high-confidence knowledge mining mechanism (KM), which selects the most similar pairs as positive pairs to increase the set of positive samples. Then, high-confidence knowledge is exchanged between different views to learn a consistent embedding distribution across views. Specifically, as shown in Figure 3, $x^{u}$ and $x^{v}\;$ are two views generated by the data x, and they are subjected to single-view contrastive learning representation (single-viewCLR) after data augmentation to obtain embeddings ${\bar{z}}^{u},\;\;{\bar{z}}^{v}$. Sets $\;S^{u},\;\;S^{v}$ are obtained by calculating the similarity between $\bar{z}$ and M by the dot product, and then mining high-confidence knowledge from the two views according to the similarity set. When the high-confidence positive samples of view v and their distribution are used to guide the learning of view u, the total loss is shown in Equation (3) [16]:

(3) $${ℒ}_{v\to u}=-log\frac{\exp\left({\bar{z}}^{u}\cdot {\hat{z}}^{u}/\tau \right)+\sum_{j\epsilon K_{+}^{v}}\exp(s_{j}^{u}s_{j}^{v})/\tau )}{\exp\left({\bar{z}}^{u}\cdot {\hat{z}}^{u}/\tau \right)+\sum_{j\epsilon K}\exp(s_{j}^{u}s_{j}^{v})/\tau )}$$

where ${ℒ}_{v\to u}$ represents the conversion of the contrast context of ${\bar{z}}^{v}$ to that of ${\bar{z}}^{u}$; $s_{j}^{u},\;s_{j}^{v}$ are the embedding contexts of $\;{\bar{z}}^{u},{\bar{z}}^{v}$.

The loss functions using two views or more are shown in Equations (4) and (5) [16]:

(4) $${ℒ}_{cross}={ℒ}_{u\to v}+{ℒ}_{v\to u}$$

(5) $${ℒ}_{cross}=\sum_{u}^{U}\sum_{v}^{U}{ℒ}_{u\to v}$$

where U is the number of views, and v≠u.
**Algorithm 2.** Main algorithm of Composite framework    **Input:** Temperature τ, momentum coefficient m, mini-batch size n, query encoder $f_{\theta_{q}}$, key encoder $f_{\theta_{k}}$, queue size K,
    **Output:** The pre-trained encoder $f_{\theta_{q}}$.
    Randomly initialize negative keys ${\left\{k_{\left(j\right)}\right\}}_{j=1}^{K}$ in queue.    **for** a sample mini-batch ${\left\{x_{\left(i\right)}\right\}}_{i=1}^{n}$ do,        **for all**
iϵ{1,⋯,n}  do                # single-view contrastive learning representation               $\;{\bar{z}}_{\left(i\right)}^{u},{\hat{z}}_{\left(i\right)}^{u}=single-viewCLR\left(x_{\left(i\right)}^{u}\right)$                ${\bar{z}}_{\left(i\right)}^{v},{\hat{z}}_{\left(i\right)}^{v}=single-viewCLR\left(x_{\left(i\right)}^{v}\right)$                # Calculate the sample similarity
               $\;S^{u}={\left\{s_{j}^{u}\right\}}_{j\in K}={\left\{{\bar{z}}_{\left(i\right)}^{u}\cdot k_{\left(j\right)}^{u}\right\}}_{j\in K}$, $\;S^{v}={\left\{s_{j}^{v}\right\}}_{j\in K}={\left\{{\bar{z}}_{\left(i\right)}^{v}\cdot k_{\left(j\right)}^{v}\right\}}_{j\in K}$                # High-confidence Knowledge Mining                 $(S_{+}^{u},K_{+}^{u})=\Gamma \left(S^{u}\right)$, $(S_{+}^{v},K_{+}^{v})=\Gamma \left(S^{v}\right)$,        **end for**        # Calculate contrastive loss ${ℒ}_{v\to u}$ for mini-batch                         ${ℒ}_{v\to u}=-\frac{1}{n}\overset{n}{\underset{i=1}{\sum }}\log\frac{\exp\left({\bar{z}}_{\left(i\right)}^{u}\cdot {\hat{z}}_{\left(i\right)}^{u}/\tau \right)+\sum_{j\epsilon K_{+}^{v}}\exp\left(s_{j}^{u}s_{j}^{v}\right)/\tau )}{\exp\left({\bar{z}}_{\left(i\right)}^{u}\cdot {\hat{z}}_{\left(i\right)}^{u}/\tau \right)+\sum_{j\in K}\exp\left(s_{j}^{u}s_{j}^{v}\right)/\tau )}$    **end for**

### 3.3. Augmentation Methods in Asymmetric Strategy

This paper uses seven data augmentation methods to learn robust action feature representations by appropriately perturbing skeleton sequences. Among the seven methods, there are four spatial augmentation methods [15]: rotation, shear, joint mask (JM), and channel mask (CM); one temporal augmentation method: crop [42]; and two spatio-temporal augmentation methods [15]: Gaussian noise (GN) and Gaussian blur (GB). We selected several typical methods for a detailed introduction.

1. Crop. In image classification tasks, crop randomly samples a part of the original image and then resizes this part to the original image size. This method is often called random cropping. For skeletons in a time sequence, some frames are firstly padded into the sequence symmetrically and then randomly cropped to the original length. The padding length is defined as T/γ, and γ is the padding ratio. This paper set γ = 6.
2. Shear. Shear augmentation is a linear transformation in the spatial dimension. Each joint is moved in a fixed direction, i.e., the shape of the 3D coordinates of body joints will be slanted with a random angle. The transformation matrix is defined as
  (6) $$A=\left[\begin{array}{ccc} 1 & a_{12} & a_{13}\\ a_{21} & 1 & a_{23}\\ a_{31} & a_{32} & 1 \end{array}\right]$$
  where $\;a_{12},a_{13},a_{21},a_{23},a_{31},$ and $a_{32}$ are the shear factors randomly sampled from [−β,β]; β is the shear amplitude, which was set to 0.5 in this paper. Then, the sequence is multiplied by the transformation matrix A in the channel dimension.
3. Gaussian blur (GB). As an effective augmentation method to reduce the level of detail and noise of images, Gaussian blur can be applied to the skeleton sequence to smooth noisy joints and decrease action details. We randomly sample σ ∈ [0.1, 2.0] for the Gaussian kernel, which is a sliding window with a length of 15. Joint coordinates of the original sequence are blurred at 50% chance by the kernel G(·) below:
  (7) $$G\left(t\right)=\exp\left(-\frac{t^{2}}{2\sigma^{2}}\right),t\in \left\{-7,-6,\cdots ,6,7\right\},$$
  where t denotes the relative position from the center skeleton, and the length of the kernel is set to 15, corresponding to the total span of t.
4. Joint mask (JM). We apply a zero-mask to a number of body joints in skeleton frames (i.e., replace all coordinates by zeros), which encourages the model to learn different local regions (i.e., except for the masked region) that probably contain crucial action patterns. To be more specific, we randomly choose a certain number of body joints (number of joints $\bar{V}\in \left\{5,6,\cdots ,15\right\}$ ) from random frames (number of frames $\bar{L}\in \left\{50,51,\cdots ,100\right\}$ ) in the original skeleton sequence to apply the zero-mask.

### 3.4. Asymmetric Data Augmentation Strategy

The advantage of contrastive learning is that it can fully learn the deep feature information of the samples. To fully learn deep features, an excellent data augmentation strategy needs to be designed to construct a robust pair of positive samples for each training sample. If multiple data augmentation methods are effectively combined to increase the difficulty of model learning, the learning effect will be significantly improved [32]. Therefore, suitable data augmentation strategies are beneficial for representation learning. However, some extreme data augmentation methods are directly used, which will make the augmented samples differ greatly from the original samples, resulting in inconsistent data representation in the self-supervised pre-training and fine-tuning stages, meaning the learned features cannot improve performance.

Based on the above problems, we propose a data augmentation strategy that combines multiple augmentation methods for skeleton sequences. This strategy combines seven data augmentation methods differently in the left and right branches of the data augmentation pipeline. Figure 1 shows the asymmetric data augmentation pipeline, where seven basic augmentation methods including crop, shear, rotation, Gaussian noise, Gaussian blur, joint mask, and channel mask are included in the left branch. Rotation, Gaussian noise, Gaussian blur, joint mask, and channel mask are randomly activated with a certain probability. Two basic augmentation methods, crop and shear, are included in the right branch.

The new data augmentation strategy is designed for the different characteristics of the three skeleton views. Crop is aimed at the temporal information of the skeleton sequence, i.e., interfering with the motion information, so it is beneficial to the augmentation of the motion view. Shear skews the 3D coordinate shape of the joint at random angles, thus changing the length of the bone, mainly for the augmentation of the bone view. The joint is the core feature information of the skeleton, which provides the 2D or 3D space coordinates of the joint point, and the remaining methods are mainly used to augment the joint view. A variety of methods directly acting on the joint view will result in a large difference between the augmented sample and the original sample, so the transformation of the joint view is performed using random probability activation. In this way, it can not only ensure that the augmentation strategy is not extreme but also make the positive sample pairs have enough differences, so that the effect of contrastive learning is more significant. Experimental results show that the network pre-trained with the asymmetric data augmentation pipeline achieves better performance in downstream tasks.

## 4. Results

Exhaustive ablation studies were conducted on NTU RGB + D 60 to examine the importance and effectiveness of different components of asymmetric data augmentation. Then, we used the linear evaluation protocol to evaluate the performance of the proposed data augmentation strategy on two large 3D skeleton datasets and compared it with similar advanced methods.

### 4.1. Dataset

The human (skeleton) action recognition datasets NTU-RGB + D 60 and NTU-RGB + D 120 proposed by the Rose Lab of Nanyang Technological University were used in this research. These two datasets both contain RGB videos, depth map sequences, 3D skeleton data, and infrared (IR) videos for each sample. We used 3D skeleton data for the study of action representation learning.

NTU-RGB + D 60 (NTU-60) [43] is a widely used and challenging large-scale dataset for action recognition tasks. The dataset contains 60 action classes with a total of 56,880 samples. There are two evaluation protocols: (1) In cross-subject (xsub), the training data come from 20 subjects, and the testing data come from another 20 subjects. The training set has 40,320 samples, and the test set has 16,560 samples. (2) In cross-view (xview), the training set and test set are divided according to the camera number. The samples collected by camera 1 are used as the test set, while the samples collected by cameras 2 and 3 are used as the training set, and the number of samples is 18,960 and 37,920, respectively.

NTU-RGB + D 120 (NTU-120) [44] is an extension of NTU RGB + D 60 and contains 113,945 skeleton sequences in 120 action classes. This dataset contains 32 setups, each denoting a specific location and background. There are two evaluation protocols: (1) In cross-subject (xsub), the training data and validation data are collected from different subjects. A total of 63,026 samples are used for training, and 50,919 samples are used for testing. (2) In cross-setup (xset), the samples with even IDs are used as the training set, while the samples with odd IDs are used as the test set, and the number of samples is 54,471 and 59,477, respectively.

### 4.2. Experimental Settings

All the experiments were conducted using the PyTorch [45] framework. Invalid frames in each skeleton sequence were first removed, and then each sequence was resized to a length of 50 frames using a linear interpolation method. The mini-batch size was set to 128. Three views were used in the experiments: joint, motion, and bone.

Data Augmentation. In the asymmetric data augmentation pipeline, we used seven augmentation methods: crop, shear, rotation, Gaussian blur, etc. In the left branch, rotation was randomly activated with a probability of 0.5, Gaussian blur, Gaussian noise, channel mask, and joint mask were activated with a probability of 0.5, and each of the four methods was activated with a probability of 0.25. In the left and right branches, crop and shear were used, and the padding ratio and shear factor were set to 6 and 0.5, respectively.

Unsupervised Pre-training. For model training, the size of the queue M in the MOCO v2 framework was set to 32,768, the momentum value was set to 0.9, and the weight decay was set to 0.0001. The model was trained for 300 epochs with a learning rate of 0.1 for the first 250 epochs and 0.01 from the 251st epoch. ST-GCN was adopted as the encoder. The encoder was trained using Equation (2) for the first 150 epochs and Equation (5) from the 150th epoch. At the same time, K = 1 was set as the default value in the knowledge mining mechanism. The detailed experimental argument setting is shown in Table 1.

Linear Evaluation Protocol. This paper followed the widely used linear evaluation protocol for linear evaluation of the action recognition task. Specifically, we trained a linear classifier (a fully connected layer followed by a softmax layer) supervised with a fixed encoder to evaluate the features learned by the model.

Performance Metrics. Top-1 accuracy: Only the action category with the highest predicted value of the model is checked. If the predicted category is the same as the label category, the prediction is correct; otherwise, the prediction is wrong. The ratio of the number of correct predictions to the total number of predictions is the Top-1 accuracy.

### 4.3. Ablation Study

Experiments were conducted on the NTU-60 dataset, following the unsupervised pre-training and linear evaluation protocol in Section 4.2, to verify the effectiveness of the asymmetric data augmentation strategy proposed in this paper.

Basic augmentation is symmetric augmentation using only two methods of crop and shear, and extreme augmentation is symmetric augmentation using seven augmentation methods simultaneously. As shown in Table 2, the accuracy using the basic augmentation strategy reached 77.8% and 83.4% on xsub and xview, respectively. After using our proposed strategy, the accuracy improved by 1.2% on both evaluation protocols. The results show that our proposed asymmetric augmentation strategy effectively improved the performance of the model. Keeping the combination strategy in the right branch unchanged, each augmentation method in the left branch of the asymmetric augmentation pipeline was removed in turn, and then the model was trained. The results show that each data augmentation method makes a corresponding contribution to the data augmentation of the skeleton. The results show that the random activation strategy of Gaussian blur, Gaussian noise, joint mask, and channel mask is the most effective augmentation method.

**Figure 4:**
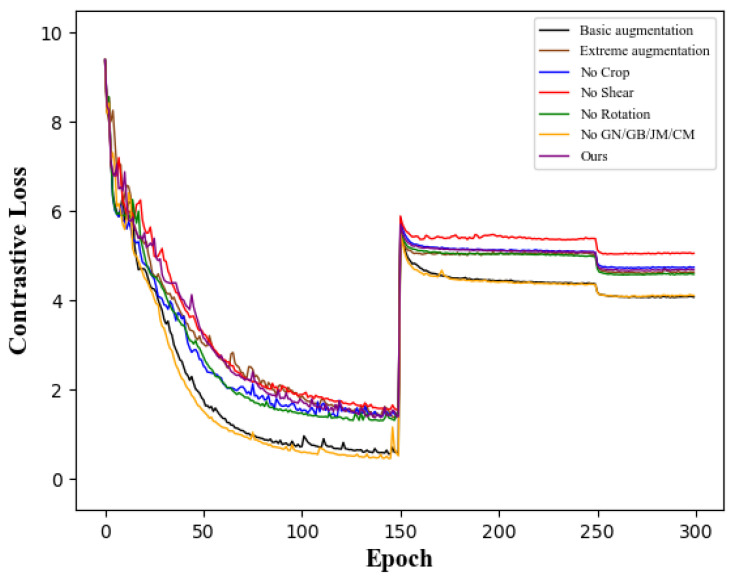
Contrastive loss curves during training using different augmentation strategies.

We plotted the model training loss curve for the joint view, as shown in Figure 4. From the training curve, it can be inferred that our method further increases the difference between pairs of positive samples in contrastive learning to prevent premature saturation of the network training loss. The network is forced to pay more attention to the dynamical commonality of skeleton sequences to learn the similarity between samples.

### 4.4. Comparison

We compared our method with other state-of-the-art methods using a linear evaluation protocol. Table 3 describes the linear evaluation results of our model at different epochs on the NTU-60 xsub dataset. Under the same training time point, our method always outperforms 3s-CrosSCLR and 3s-SkeletonCLR. At 100 epochs, it even achieves the same results as 3s-SkeletonCLR at 300 epochs, which shows that our method can effectively improve the performance of the model.

Linear Evaluation Results on NTU-60. As shown in Table 4, our method outperforms all other methods [16,37,46], leading 3s-SkeletonCLR by 4.0% and 4.8% under the xsub and xview protocols, respectively. The results show that good data augmentation enables the model to learn better feature representations, thereby improving the recognition accuracy.

Linear Evaluation Results on NTU-120. As shown in Table 5, our method outperforms other self-supervised methods on NTU-120, leading 3s-CrosSCLR by 0.6% and 3.2% under the xsub and xset protocols, respectively, achieving 68.5% and 69.9% accuracy. The results show that our method is also competitive on multi-class, large-scale skeleton action recognition datasets.

The experimental results show that the model trained by the proposed asymmetric data augmentation strategy achieved remarkable results on two large-scale skeleton action recognition datasets, NTU-60 and NTU-120, further validating the effectiveness of our proposed method. At the same time, this shows that the design of the data augmentation method plays a very important role in the effect of self-supervised learning.

## 5. Conclusions and Future Work

This paper proposes an asymmetric data augmentation strategy to appropriately transform skeleton data to explore new motion patterns. Multiple data augmentation methods are used in combination to increase the difficulty of learning a contrastive learning model to learn high-quality action representations. A widely used linear evaluation protocol was used to verify the effectiveness of our method. Our method achieved 79.0% and 84.6% Top-1 recognition accuracy on the two evaluation protocols of NTU-RGB + D 60. The Top-1 performance indicators of 68.5% and 69.9% were obtained on the two evaluation protocols of NTU-RGB + D 120. Compared with other methods from the same type of research, the performance is improved. The results show that the proposed asymmetric data augmentation strategy is effective for skeleton-based action representation learning.

However, there are still some limitations of our method. First, we only augmented the three selected skeleton views, which enriched the skeleton information to some extent, but more views may mean better results. Second, we only used the basic contrastive learning framework and feature extraction network and did not explore the performance improvement that more advanced methods may bring. Future work includes the following: a higher-performance contrastive learning framework and feature extraction network will be studied and used to learn good feature representations; more skeleton views will be selected to further enrich skeleton information and make the learned feature representation more robust.

## Figures and Tables

**Table 1:** Experimental arguments setting.

Arguments	Value
sequence size	50 frames
batch size	128
view	joint, motion, bone
base encoder	st-gcn
queue size	32,768
momentum	0.9
weight decay	0.0001
epoch	300
learning rate	0.1(before 250 epoch)|0.01(after 250 epoch)
loss function	Equation (2) (before 150 epoch)|Equation (5) (after 150 epoch)
knowledge mining	1
padding ratio	6
shear factor	0.5

**Table 2:** Ablation experiments for data augmentation strategy on NTU-60 dataset.

Asymm. Aug.	Augmentations	xsub (%)	xview (%)
×	Basic augmentation	77.8	83.4
×	Extreme augmentation	76.5	83.1
√	No Crop	77.7	82.3
√	No Shear	77.3	83.6
√	No Rotation	78.8	84.3
√	No GN/GB/JM/CM	76.3	81.9
√	Ours	79.0	84.6

**Table 3:** Linear evaluation results on NTU-60 xsub for different epochs.

Method	100 ep	150 ep	200 ep	300 ep
3s-SkeletonCLR [16]	71.3	73.8	74.1	74.1
3s-CrosSCLR [16]	70.0	72.8	76.0	77.2
ours	74.1	76.0	77.9	79.0

**Table 4:** Linear evaluation results on NTU-60 dataset.

Method	xsub (%)	xview (%)
3s-SkeletonCLR [16]	75.0	79.8
3s-Colorization [46]	75.2	83.1
3s-CrosSCLR [16]	77.8	83.4
3s-AimCLR [37]	78.9	83.8
ours	79.0	84.6

**Table 5:** Linear evaluation results on NTU-120 dataset.

Method	xsub (%)	xset (%)
P&C [11]	42.7	41.7
AS-CAL [15]	48.6	49.2
3s-CrosSCLR [16]	67.9	66.7
ISC [38]	67.9	67.1
3s-AimCLR [37]	68.2	68.8
ours	68.5	69.9

## Data Availability

Not applicable.
